# Sexual dysfunction, depression, and the impact of antidepressants

**DOI:** 10.1192/j.eurpsy.2024.1120

**Published:** 2024-08-27

**Authors:** T. Jupe, I. Giannopoulos, A. Roumpou

**Affiliations:** Psychiatric Hospital of Attica, Athens, Greece

## Abstract

**Introduction:**

Sexual dysfunction is a common side effect of antidepressants and can have significant impact on the person’s quality of life, relationships, mental health, and recovery. The reported incidence of sexual dysfunction associated with antidepressant medication varies considerably between studies, making it difficult to estimate the exact incidence or prevalence.

**Objectives:**

The focus of this e-poster is to explore the incidence, pathophysiology, and treatment of depression disorder and antidepressant iatrogenic sexual dysfunction.

**Methods:**

A bibliopgraphical review was performed using PubMed platform. All relevant articles were found using the keywords: depression, sexual disfunction, antidepressant.

**Results:**

Sexual dysfunction is a common symptom of depression. Although decreased libido is most often reported, difficulties with arousal, resulting in vaginal dryness in women and erectile dysfunction in men, and absent or delayed orgasm are also prevalent. Sexual dysfunction is also a frequent adverse effect of treatment with most antidepressants and is one of the predominant reasons for premature drug discontinuation. Selective serotonin reuptake inhibitors are the most widely prescribed antidepressants and have significant effects on arousal and orgasm compared with antidepressants that target norepinephrine, dopamine, and melatonin systems. The availability of an antidepressant that does not cause or exacerbate sexual dysfunction represents an advance in pharmacotherapy for mood disorders and should reduce treatment noncompliance and decrease the need for switching antidepressants.

**Conclusions:**

The sexual problems reported range from decreased sexual desire, decreased sexual excitement, diminished or delayed orgasm, to erection or delayed ejaculation problems. There are a number of case reports of sexual side effects, such as priapism, painful ejaculation, penile anesthesia, loss of sensation in the vagina and nipples, persistent genital arousal and nonpuerperal lactation in women.

**Disclosure of Interest:**

None Declared

